# Protective Mechanisms of Guanosine from *Solanum lycopersicum* on Agonist-Induced Platelet Activation: Role of sCD40L

**DOI:** 10.3390/molecules18078120

**Published:** 2013-07-10

**Authors:** Eduardo Fuentes, Marcelo Alarcón, Luis Astudillo, Claudio Valenzuela, Margarita Gutiérrez, Iván Palomo

**Affiliations:** 1 Department of Clinical Biochemistry and Immunohematology, Faculty of Health Sciences, Interdisciplinary Excellence Research Program on Healthy Aging (PIEI-ES), Universidad de Talca, Talca 3460000, Chile; 2 Centro de Estudios en Alimentos Procesados (CEAP), CONICYT-Regional, Gore Maule, R09I2001, Talca 3460000, Chile; 3 Synthesis Laboratory, Chemical Institute of Natural Resources, Universidad de Talca, Talca 3460000, Chile; 4 Institute of Plant Biology and Biotechnology, Universidad de Talca, Talca 3460000, Chile

**Keywords:** guanosine, antiplatelet, sCD40L, *Solanum lycopersicum*, functional foods

## Abstract

In the past 30 years, only three natural products have been sources of new drugs with antiplatelet activity. In this study, we have demonstrated for the first time that guanosine from *Solanum lycopersicum* possesses antiplatelet (secretion, spreading, adhesion and aggregation) activity *in vitro* and inhibition of platelet inflammatory mediator of atherosclerosis (sCD40L). According to ADP-induced platelet aggregation inhibiting, the total extract residue was fractionated by liquid chromatography/phase separation, affording an aqueous fraction. This fraction was subjected to repeated permeation over Sephadex LH-20 and semi-preparative TLC. The isolated compound finally obtained was identified as guanosine on the basis of its UV-spectra, HPLC and ^1^H-NMR data. Guanosine concentration dose-dependently (1 to 4 mmol/L) inhibited platelet secretion and aggregation induced by ADP and collagen. Spread of human platelets on collagen in the presence of guanosine was fully inhibited. After incubation of whole blood with guanosine, the platelet adhesion and aggregation under flow conditions was inhibited concentration dependently (0.2 to 2 mmol/L). At the same concentrations that guanosine inhibits platelet aggregation, levels of sCD40L were significantly decreased. Guanosine is thus likely to exert significant protective effects in thromboembolic-related disorders by inhibiting platelet aggregation.

## 1. Introduction

The incidence and prevalence of cardiovascular diseases (CVD) (*i.e*., acute myocardial infarction, cerebrovascular disease and peripheral arterial thrombosis) has increased significantly in recent years, at least in part as a result of the progressive aging of the population [1,2]. According to the World Health Organization, by 2020 CVD will represent about 30% of deaths worldwide [3], with a relative increase over time due to the aging of the population [4].

In most cases, myocardial infarction and stroke are the consequence of atherosclerotic plaque rupture and thrombus formation. The current lifestyle of the population contributes to the development of risk factors for CVD, such as hypertension, diabetes, smoking, and hypercholesterolemia [5,6]. The development and progression of CVD lies in the interactive processes of atherosclerotic lesions and thrombus formation, an interaction established primarily by platelet participation [7]. 

Circulating platelets in peripheral blood play a major role in maintaining blood homeostasis [8]. However, following an atheromatous plaque rupture, platelets adhere, secrete their granule contents, aggregate and initiate cardiovascular disorders such as thrombosis [9]. It has also been demonstrated that platelets are involved not only in the arterial thrombotic process, but that they also play an active role in the inflammatory process of atherogenesis [10]. Reports in the last decade have described the secretion by platelets of pro-inflammatory molecules that exacerbate the inflammatory response in the atherosclerotic plaque (sCD40L) [10]. Thus, platelets are the main source of the soluble CD40 ligand (sCD40L), contributing to 95% of its circulating levels [11]. High levels of sCD40L significantly increase platelet activation and aggregation, while blockade of this pathway with anti-CD40L antibodies can prevent or delay atheroinflammation progression [12].

The inhibition of the platelet function has been used for long time in an effort to prevent the ischemic complications at late stages of the atherosclerotic process, which are the leading causes of cardiovascular morbidity and mortality [13]. 

From the point of view of public health, efforts should be directed to primary prevention, namely, to reduce the aforementioned cardiovascular risk factors [14,15]. In this context, regular consumption of fruits and vegetables (F&V), part of the so called Mediterranean Diet [16], has been shown to be beneficial in terms of reducing the incidence of CVD in certain populations. This effect might be mediated by bioactive principles present in F&V [17] which explains the increasing attention focused in exploring phytochemicals in the prevention of CVD [18]. Nevertheless, in the last 30 years only three such phytochemicals have generated new drugs derived from natural products with platelet aggregation inhibition and only six with antithrombotic activity [19]. In this sense, highly consumed tomato (*Solanum lycopersicum*) also presents antiplatelet activity [20]. In addition, our group recently isolated and identified adenosine from *S. lycopersicum*. Adenosine showed a potent antiplatelet activity through the inhibition of platelet secretion, adhesion and aggregation [21]. The purpose of this research was to isolate and identify bioactive compounds from *S. lycopersicum* that present antiplatelet activity and inhibition of platelet sCD40L release.

## 2. Results and Discussion

### 2.1. Bioassay-Guided Isolation of Antiplatelet Compound

*S. lycopersicum*, fresh or processed (e.g., tomato paste), apart from their nutritional value, has been found to provide a cardioprotective effect, both at the endothelial and platelet levels [22]. Aqueous and methanol total extracts from *S. lycopersicum* were found to be thermally stable in the temperature range of 20 to 100 °C and neither acid nor alkali affected inhibition of platelet aggregation induced by ADP [20].

**Figure 1:**
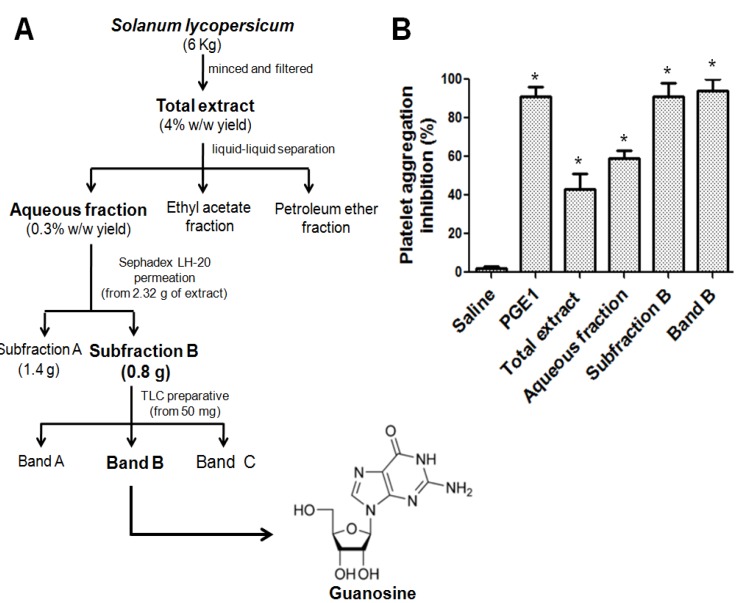
Scheme of the extraction and bioguided fractionation of *S. lycopersicum*. (**A**) Bio-directed isolation of band B-guanosine. (**B**) Platelet antiaggregant activity of extract and fractions from *S. lycopersicum.* Platelet aggregation was induced by ADP 8 µmol/L and all samples at 1 mg/mL. Saline (negative control) and PGE_1_ (positive control). The graph depicts the average ± SEM of n = 3 experiments. All values are statistically significant *vs*. saline (* *p* < 0.05).

The scheme of the extraction and bioguided fractionation by platelet antiaggregant activity of *S. lycopersicum* is presented in Figure 1.

After of the liquid chromatography/phase separation, the aqueous fraction (0.3% w/w yield) had more platelet antiaggregant activity than total extract, and ethyl acetate and petroleum ether fractions. Thus considering platelet aggregation induced by ADP, the inhibition was in the following order: aqueous (54 ± 13%, *p* < 0.05), petroleum ether (43 ± 6, *p* < 0.05) and ethyl acetate (39 ± 8, *p* < 0.05) fractions. To advance in the isolation and identification of bioactive compound with antiplatelet activity, the aqueous fraction was subjected to repeated permeation over Sephadex LH-20 and 21 fractions of 17 mL each were collected. The fractions were monitored at 254 nm and two sub fractions were identified by HPLC (sub-fractions A and B). While platelet aggregation induced by ADP in the presence of sub-fraction A (1 mg/mL) was inhibited by 78 ± 11% (*p* < 0.05), the platelet aggregation induced by ADP was completely inhibited by sub-fraction B at 1 mg/mL (92 ± 7%, *p* < 0.05), so further purification was carried out. Sub-fraction B (50 μg by plate) was thus subjected to semi-preparative TLC. Under UV light (254 nm) three bands (A, B and C) were observed, removed, and extracted with methanol. Since platelet aggregation induced by ADP was inhibited by band B at 1 mg/mL by 95 ± 5%, further identification of this band was carried 0ut (Figure 1). 

### 2.2. Identification of the Antiplatelet Compound

Band B was identified as guanosine according to the UV spectrum (λ_max_ = 219 and 258 nm) and a HPLC retention time similar to that of a guanosine standard (Rt = 2.8 min). The structure was confirmed by NMR spectroscopy, whereby the ^1^H-NMR spectrum of Band B was consistent with the structure of guanosine, the data obtained was also consistent with a previous report [23].

Based on HPLC determination, the content of guanosine in extracts from *S. lycopersicum* was in the following order: skin extract > pulp extract. Such results were calculated from a guanosine standard linear regression with a correlation coefficient of r = 0.9478. The skin extract showed the highest content of guanosine (3.8 mg/g dried extract), while tomato pulp showed the lowest amount (1.6 mg/g dried extract). The content of guanosine (mg/g dried extract) is about 50-fold less than that of adenosine in tomato pulp extract [21].

### 2.3. Effects of Guanosine on Platelet Function

The inhibition of platelet function has been used for long time in an effort to prevent and treat CVD [13], but the morbidity and mortality figures, however, indicate that current anti-platelet strategies (and anti-coagulant therapy) are far from a panacea [24]. Chemically synthesized antiplatelet drugs are even frequently associated with serious adverse effects (internal bleeding and gastrointestinal adverse effects, among others) [24]. Moreover, epidemiological studies have provided evidence of a protective role of healthy diets in the prevention of CVD [20]. In this context, the beneficial effects of F&V could be related to the bioactive principles found in them [25,26]. In addition, our group recently isolated and identified adenosine from *S. lycopersicum.* Adenosine at a low concentration showed a potent antiplatelet activity through the inhibition of platelet secretion, adhesion and aggregation [21]. 

**Figure 2:**
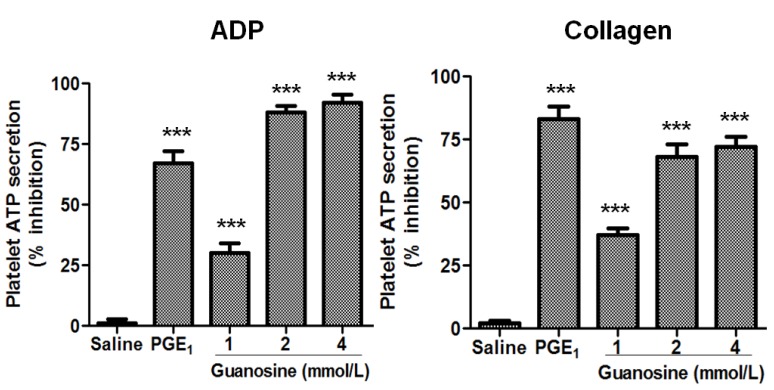
Guanosine concentration-dependently inhibited platelet ATP secretion. Effect of guanosine on ADP (8 µmol/L) and collagen (1.5 μg/mL) induced platelet ATP secretion. Saline (negative control) and PGE_1_ (positive control). Results were expressed as % inhibition (mean ± SEM, n = 3).

**Figure 3:**
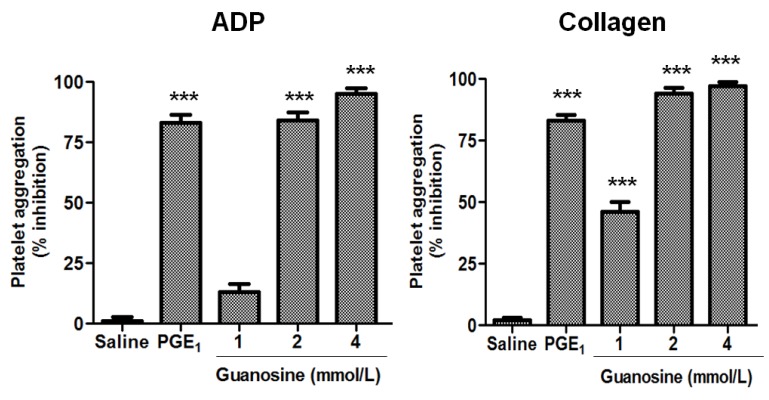
Guanosine concentration-dependently inhibited platelet aggregation. Quantitation of the inhibitory effect of guanosine on platelet aggregation induced by ADP (8 µmol/L) and collagen (1.5 μg/mL). Saline (negative control) and PGE_1_ (positive control). Results were expressed as % inhibition (mean ± SEM, n = 3).

The effects of guanosine on platelet ATP secretion and aggregation were studied. First, the effect of guanosine on platelet secretion induced by ADP and collagen is presented in Figure 2. The human platelet ATP secretion induced by ADP in the presence of guanosine 1, 2 and 4 mmol/L was inhibited by 30 ± 7, 88 ± 5 and 92 ± 6% (*p* < 0.001), respectively, while platelet ATP secretion induced by collagen was inhibited by 37 ± 5, 68 ± 9 and 72 ± 7% (*p* < 0.001) at concentrations of 1, 2 and 4 mmol/L. Moreover, guanosine concentration-dependently (1, 2 and 4 mmol/L) inhibited human platelet aggregation stimulated by ADP and collagen (Figure 3). 

Thus, the platelet aggregation induced by ADP in the presence of guanosine 2 and 4 mmol/L was inhibited by 84 ± 6 and 95 ± 4% (*p* < 0.001), respectively, while platelet aggregation stimulated by collagen was inhibited by 46 ± 7, 94 ± 4 and 97 ± 3% (*p* < 0.001) at concentrations of 1, 2 and 4 mmol/L, respectively. The antiplatelet effect of guanosine was demonstrated with the use of various agonists (ADP and collagen). These results also indicate that the site of action of guanosine is not at the receptor level of individual agonists, because of guanosine-inhibited platelet secretion and aggregation induced by ADP and collagen. Moreover, one possible mechanism to inhibit two different platelet receptors is by increasing levels of cAMP. Of this form cAMP downregulates P2Y1R expression [27] and maintains GPVI in a monomeric form, thus keeping platelets in a resting state [28].

### 2.4. Inhibitory Effect of Guanosine on Platelet Spreading on Immobilized Collagen

**Figure 4:**
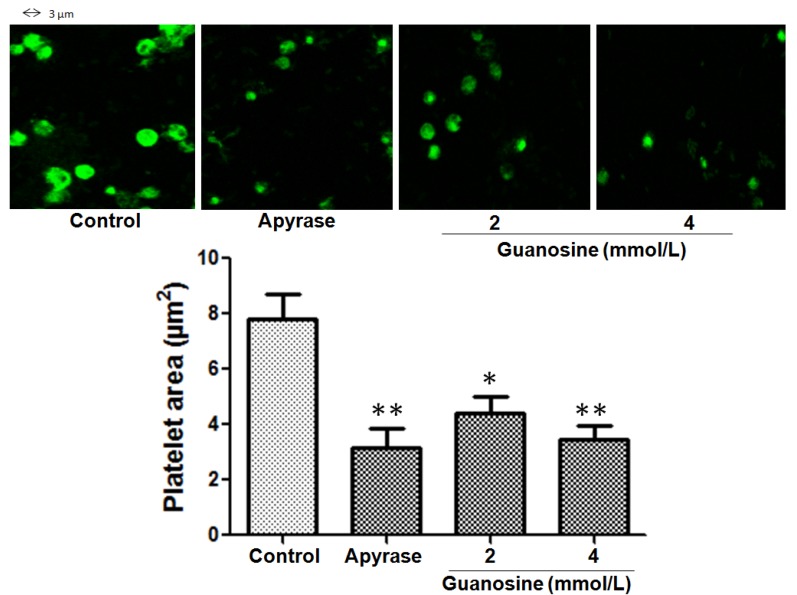
Effect of guanosine on spreading of human platelets on collagen-coated surfaces. Platelet area (µm^2^) mean ± SEM was acquired from 4 consecutive fields using a Carl Zeiss LSM 700 confocal microscope. Control corresponds to saline: maximum spread. * *p* < 0.05 and ** *p* < 0.01.

When platelets adhere to collagen, a ligand-binding–induced signal is generated, leading to platelet spreading that render adherent platelets resistant to the shear forces at the site of vascular damage [29]. To study whether guanosine was able to inhibit spreading (platelet shape change), platelets were allowed to adhere to collagen and spreading was evaluated by differential interference contrast and confocal microscopy. The fuly spreading (platelet surface area) of human platelets on immobilized collagen in the presence of guanosine 2 and 4 mmol/L was inhibited from 7.8 ± 1 to 4.4 ± 1 and 3.3 µm^2^ (platelet area), respectively (*p* < 0.05) (Figure 4). 

### 2.5. Guanosine Reduces Platelet Adhesion and Aggregation under Flow Conditions

**Figure 5:**
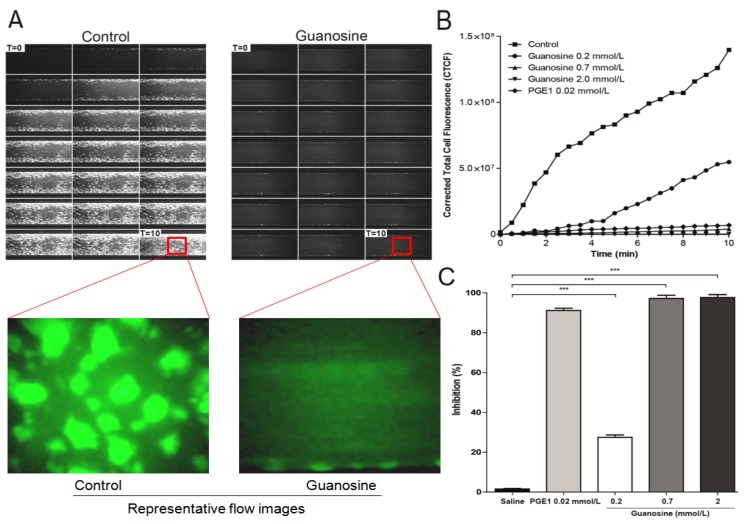
Effect of guanosine on collagen-induced platelet adhesion and aggregation under arterial flow conditions. Citrate-anticoagulated blood was pre-incubated with saline (control), PGE_1_ (0.02 mmol/L) or guanosine (0.2 to 2 mmol/L) for 1 hour and then was perfused over plaque-coated surfaces for 10 min at room temperature at a shear rate of 1,000 s^−1^. (**A**) timelapse of 10 min at 1,000 s^−1^, at 30 s intervals, guanosine (2 mmol/L). (**B**) It shows the intensity (CTCF) over a time lapse. (**C**) bar diagram (values are mean ± SEM; n = 3). *** *p* < 0.001.

The platelet adhesion cascade takes place in the presence of shear flow, a factor not accounted for in conventional (static) well-plate assays. Under conditions of arterial shear, platelets tethered to immobilized collagen [29]. This response in pathological conditions cause vascular alterations and blood flow disturbances (arterial occlusion), most frequently in atherosclerotic vessels of the heart and brain [30]. The effects of guanosine (0.2 to 2 mmol/L) on platelet adhesion/aggregation to immobilized collagen under arterial flow conditions are shown in Figure 5. After perfusion of citrate-anticoagulated blood over collagen coated plaque surfaces at 37 °C with a wall shear rate of 1000 s^−1^ for 10 min, rapid platelet adhesion and aggregate formation were observed. Guanosine concentration-dependently (0.2 to 2 mmol/L) reduced collagen-induced platelet adhesion and aggregate formation under controlled flow, with IC_50_ value of 0.45 mmol/L (*p* < 0.001) (Figure 5). Meanwhile, PGE_1_ (0.02 mmol/L) presented an inhibition of 89 ± 4% (*p* < 0.05).

### 2.6. Inhibitory Effect of Guanosine on Levels of sCD40L

**Figure 6:**
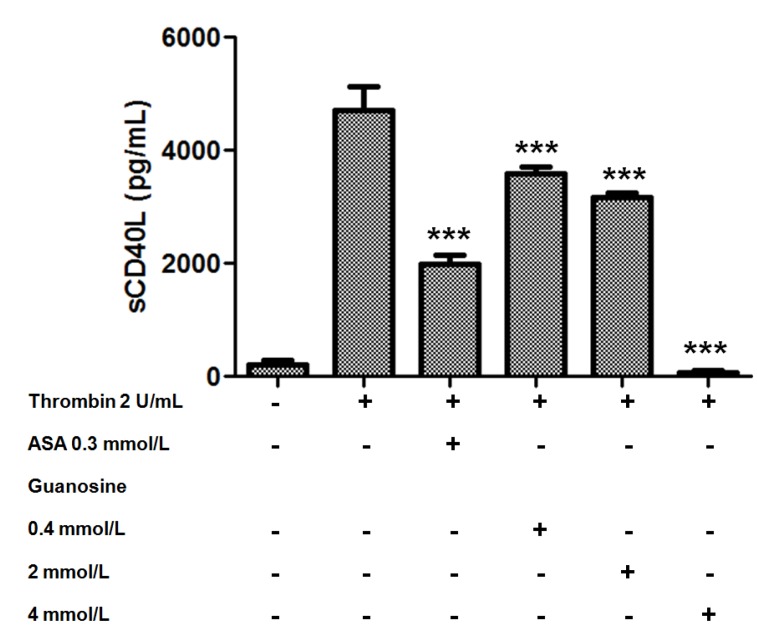
Effect of guanosine on release of sCD40L from human platelets induced by thrombin. Washed platelets were pretreated with saline, ASA, acetylsalicylic acid 0.3 mmol/L or guanosine (0.4 to 4 mmol/L) for 15 min at 37 °C and then stimulated by thrombin (2 U/mL). The graph depicts the mean ± SEM of n = 3 experiments. *** *p* < 0.001.

In the last decade it has been described that the secretion of platelet pro-inflammatory molecules (sCD40L, RANTES, sP-selectin, among others) may play a pathogenic role in both the long-term atherosclerotic process, and in the triggering and propagation of acute coronary syndromes [31,32]. CD40L-CD40 interaction between platelet-leukocyte may play a pathogenic role in atherosclerotic progress [31]. sCD40L is released from platelets and contributes to various steps in atherosclerotic lesion progression: inflammation, thrombosis and restenosis [33]. Thus, high levels of sCD40L have been associated with platelet activation, suggesting a prognostic value in patients with advanced atherosclerosis [34]. As platelets are considered the major source of sCD40L in the blood and sCD40L plays a pivotal role in atherosclerosis [35], we examined the effect of guanosine on release of sCD40L. As observed in figure 6, we found that guanosine significantly reduced thrombin-induced sCD40L release from platelets. Thus, guanosine attenuated the effect of thrombin-induced sCD40L release by 24 ± 4, 33 ± 2 and 98 ± 1% (*p* < 0.001) at concentrations of 0.4, 2 and 4 mmol/L, respectively (Figure 6).

**Figure 7:**
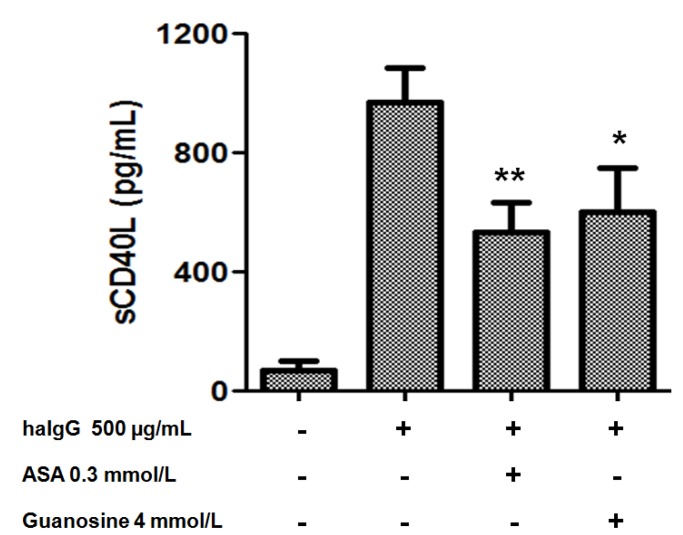
Effect of guanosine on release of sCD40L from platelets induced by haIgG. Washed platelets were pretreated with saline, ASA, acetylsalicylic acid 0.3 mmol/L or guanosine (4 mmol/L) for 15 min at 37 °C and then stimulated by haIgG (500 µg/mL). The graph depicts the mean ± SEM of n = 3 experiments. * *p* < 0.05, ** *p* < 0.01.

Furthermore, platelets have been reported to respond to IgG-complexes by release of inflammatory mediator sCD40L. In this study, guanosine at 4 mmol/L was able to inhibited platelet sCD40L release by 38 ± 2% (*p* < 0.05) (Figure 7). This study demonstrates for the first time that guanosine from *S. lycopersicum* not only presents antiplatelet activity, but also decreases the inflammatory component of activated platelets, through a lower release of sCD40L. This inhibition of sCD40L can prevent the onset of an atherosclerotic lesion [36].

## 3. Experimental

### 3.1. Chemicals and Reagents

HPLC grade acetonitrile from Merck (Darmstadt, Germany) was used. Sephadex LH-20 (Pharmacia Fine Chemicals, Piscataway, NJ, USA) and thin layer chromatography (TLC) plates (Merck, Darmstadt, Germany) were used for chromatography. Guanosine, calcein-AM and human IgG were purchased in Sigma-Aldrich (St. Louis, MO, USA). Sodium chloride (p.a.), formic acid, petroleum ether, methanol, ethyl acetate were obtained from Arquimed (Santiago, Chile). The agonists adenosine 5'- diphosphatebis (ADP) and collagen, bovine serum albumin (BSA), acetylsalicylic acid (ASA) and prostaglandin E1 (PGE_1_) were also obtained from Sigma-Aldrich, whereas Luciferase/ luciferin reagent was obtained in Chrono-Log corp (Havertown, PA, USA) and microfluidic chambers were from Bioflux (Fluxion, San Francisco, CA, USA). 

### 3.2. Processing Material

Fresh tomatoes (H9665, H9997 and H7709) were obtained from the “Sugal Chile” (before “Tresmontes Luchetti”, production plant Talca, Chile).

### 3.3. Bioassay-Guided Isolation of Bioactive Compound

The aqueous extract residue was fractionated by liquid chromatography /phase separation, obtaining petroleum ether, ethyl acetate and aqueous fractions. Since the aqueous fraction had significant antiplatelet properties further purification was carried out. The aqueous fraction was lyophilized (Freezone 6, Labconco, Kansas City, MO, USA).

With the goal of isolating and identifying one of the bioactive compounds from *S. lycopersicum* the aqueous fraction was subjected to repeated permeation over Sephadex LH-20 (column length 60 cm, internal diameter 3 cm) using MeOH-H_2_O 4:1 as eluent. Semi-preparative TLC was performed on 10 cm × 20 cm TLC silica gel plates coated with 1 mm layer and the sample was applied. The plate was developed using 25 mL of organic mobile phase EtOAc-AcOH-H_2_O 10:2:3 v/v/v in a saturated chamber. The plate under UV light (254 nm) was developed. The bands observed were removed, extracted with methanol and lyophilized. Since one of the bands had significant antiplatelet properties further identification was carried out.

### 3.4. Chemical Identification of Antiplatelet Compound

#### 3.4.1. Spectral Scanning

Spectral scanning between 200 and 600 nm was used to investigate the UV-visible maximum band absorption. The UV/Vis spectra were obtained in a spectrophotometer (Unicam, Cambridge, UK) using MeOH as a solvent.

#### 3.4.2. NMR Analysis

The structure of the bioactive band was determined by ^1^H-NMR on a Bruker AMX spectrometer (Bruker, Germany) operating at 400 MHz, using DMSO-D_6_ as solvent. TMS was used as an internal standard. Chemical shifts (δ) and *J* values were reported in ppm and Hz, respectively, relative to the solvent peak (DMSO-D_6_ at 2.500 and 3.257 ppm for protons). Signals were designated as follows: s, singlet; d, doublet; dd, doublet of doublets; t, triplet and m, multiplet. Data was as follows: δ: 10.571 (1H, s); 7.910 (1H, s); 6.393 (2H, s); 5.696 (1H, d, *J* = 6 Hz); 5.300 (1H, d, *J* = 6 Hz); 5.031 (1H, s); 4.962 (1H, s); 4.399 (1H, d, *J* = 5.2 Hz); 4.097 (1H, s); 3.881 (1H, s); 3.577 (2H, m). 

#### 3.4.3. HPLC Analysis

Analyses of the chemical profile of the bioactive band was performed by an HPLC Merck-Hitachi (La-Chrom, Tokyo, Japan) equipment consisting of an L-7100 pump, an L-7455 UV diode array detector and a D-7000 chromatointegrator. A UV spectrum from 200 to 600 nm was recorded for peak characterization. HPLC–DAD analysis was carried out using a 250 mm × 4.60 mm i.d., 5 µm C18-RP Luna column (Phenomenex, Torrance, CA, USA) maintained at 25 °C with a linear gradient solvent system consisting of 1% formic acid (A) and acetonitrile (B) as follows: 95%–90% A over 10 min; 90%–50% A over 10 min; 50%–0% A over 5 min and followed by 0%–95% A from 25 to 30 min at a flow rate of 1 mL/min. Standard of guanosine used for HPLC–DAD identification, was dissolved in acetonitrile–formic acid (99:1, v/v) to prepare standard solutions ranging from 0.125 to 1 mg/mL. Extracts (pulp and skin) from *S. lycopersicum* were lyophilized and then equilibrated to room temperature for 1 h and dissolved in acetonitrile–formic acid (99:1, v/v). The samples were filtered through a 5 µm filter (Millipore Corporation, Bedford, MA, USA) and then 20 µL were injected. All samples were run in triplicate. 

### 3.5. Preparation of Human Platelet Suspensions

Venous blood samples were taken from two volunteers (healthy university students, who previously signed informed consent) in 3.2% citrate tubes (9:1 v/v) by phlebotomy with vacuum tube system (Becton Dickinson Vacutainer Systems, Franklin Lakes, NJ, USA). The protocol was authorized by the ethic committee of Universidad de Talca in accordance with the Declaration of Helsinki (approved by the 18th World Medical Assembly in Helsinki, Finland, 1964). The samples were gently homogenized by 5-fold inversion and allowed to stand for 5 min. The tubes were centrifuged (DCS-16 Centrifugal Presvac RV) at 240 *g* for 10 min to obtain platelet-rich plasma (PRP). Platelet count in PRP was performed in a hematologic counter (Bayer Advia 60 Hematology System, Tarrytown, NY, USA). The original tubes were centrifuged at 650 g for 10 min to obtain the platelet-poor plasma (PPP). Finally, the PRP was adjusted to 200 × 10^9^ platelets/L with PPP. Washed platelets were prepared by adding 50 ng/mL PGE_1_ to the PRP and platelets were then pelleted at 750 g for 10 min. Platelets were washed once in Tyrode-HEPES buffer containing 50 ng/mL PGE_1_ and 1 mmol/L EDTA, pH 7.4, and resuspended in Tyrode-HEPES to a concentration of 200 × 10^9^ platelets/L.

### 3.6. Measurement of Platelet Secretion

Platelet secretion was determined by measuring the release of ATP using luciferin/luciferase reagent [21]. Luciferin/luciferase (50 μL) was added to 480 μL of platelet suspension (PRP adjusted to 200 × 10^9^ platelets/L) within 2 min before stimulation. Platelet secretion was measured for 6 min in real time in a lumi-aggregometer (Chrono-Log, Havertown, PA, USA) at 37 °C with stirring (150 g). To examine the effects on platelet secretion, platelets were preincubated with 20 µL of saline (negative control), PGE_1_ (0.02 mmol/L, positive control) or guanosine (1, 2 and 4 mmol/L) for 3 min prior to the addition of ADP (8 µmol/L) or collagen (1.5 μg/mL). The inhibition of the maximal platelet secretion by guanosine was expressed as a percentage with respect to control (saline): 100 − ((X × 100)/Y) (X: result of the component under study and Y: result of the negative control, saline).

### 3.7. Platelet Spreading Assay

Coverslips were coated with collagen (100 μg/mL) and incubated at 37 °C for 60 min. Then were rinsed with phosphate buffer saline (PBS), blocked with BSA 1% for 60 min at 37 °C and finally washed with PBS to remove any unbound BSA. Washed platelets (5 × 10^9^ platelets/L) were labeled with calcein-AM and preincubated with saline (negative control), apyrase (positive control, 2 units/mL) or guanosine (2 and 4 mmol/L) for 1 hour at room temperature and then allowed to spread on collagen for 1 hour at 37 °C [37]. After gently rinsing 3 times with PBS, spread platelets were mounted in Vectashield mounting medium (Vector Laboratories, Burlingame, CA, USA). The images were acquired with Zeiss 40x oil-immersion lens (1.3 numeric aperture) and Photometrics SenSys camera (Photometrics, Tucson, AZ, USA) from 4 consecutive fields, examined using a Carl Zeiss LSM 700 confocal microscope (Carl Zeiss, Oberkochen, Germany) and the argon–krypton laser at 488 nm to generating the fluorescent calcein signal detected between 490 nm and 530 nm. The images were analyzed using Image J software (version 1.26t, NIH, Stapleton, New York, NY, USA).

### 3.8. Measurement of Platelet Aggregation

Platelet aggregation was monitored by light transmission according to Born and Cross [38], using a lumi-aggregometer. Briefly PRP (480 μL of PRP adjusted to 200 × 10^9^ platelets/L) in the reaction vessel were pre-incubated with saline (20 μL, negative control), PGE_1_ (0.02 mmol/L, positive control), extracts or fractions (1 mg/mL), or guanosine (1, 2 and 4 mmol/L). After 3 min of incubation agonist (20 μL) was added to initiate platelet aggregation, which was measured for 6 min. ADP (8 µmol/L) and collagen (1.5 μg/mL) were used as agonists. All measurements were performed in triplicate. The results of platelet aggregation (maximal amplitude [%], slope, area under the curve and lag time [s]) were determined by the AGGRO/LINK software (Chrono-Log). The inhibition of the maximal platelet aggregation by guanosine was expressed as a percentage with respect to control (saline): 100 − ((X × 100)/Y) (X: result of the component under study and Y: result of the negative control, saline).

### 3.9. Analysis of Platelet Adhesion and Aggregation under Controlled Flow

For flow experiments, a BioFlux 200 flow system (Fluxion) with high shear plates (48 wells, 0–20 dyne/cm^2^) was used. Using manual mode in the BioFlux software, the microfluidic chambers were coated for 1 hour with 20 μL of collagen 200 µg/mL at a wall shear rate of 200 s^−1^. 

The plaque coating was allowed to dry at room temperature for one hour. The channels were perfused with PBS for 10 min at room temperature a wall shear rate of 200 s^−1^ for removing the interface. Then, the channels were blocked with BSA 5% for 10 min at room temperature a wall shear rate of 200 s^−1^. In order to visualize platelets, the citrate-anticoagulated blood containing calcein-AM (4 μmol/L) was added to the inlet well, and chambers were perfused for 10 min at room temperature a wall shear rate of 1,000 s^−1^.

The plaque-coated micro-fluidic high shear plates were mounted on the stage of an inverted fluorescence microscope (TE200, Nikon, Tokyo, Japan). Whole blood with saline, PGE_1_ (0.02 mmol/L) or guanosine (0.2 to 2 mmol/L) was pre-incubated at room temperature for 1 hour prior to the start of flow, and experiments were performed at room temperature [39].

Platelet deposition was observed and recorded in real-time (30 frames per min) with a CCD camera (QICAM, QIMaging, Surrey, BC, Canada). We used bright field and fluorescence microscopy for real-time visualization of platelet adhesion and aggregation in flowing blood. For each flow experiment, fluorescence images were later analyzed off-stage by quantifying the area covered by platelets with the ImageJ software (version 1.26t, NIH, USA). In each field, the areas covered by platelets were quantified. The inhibition of platelet adhesion and aggregation under flow conditions was expressed as a percentage with respect to control (saline): 100 − ((X × 100)/Y) (X: result of the component under study and Y: result of the negative control, saline).

### 3.10. Measurement of sCD40L Levels

sCD40L was determined using a Human sCD40-Ligand Quantikine kit (R&D Systems, Minneapolis, MN, USA). Briefly, washed platelet was pretreated with saline (negative control), ASA (0.3 mmol/L, positive control) or guanosine (0.4 to 4 mmol/L) for 15 min at 37 °C and then stimulated by thrombin (2 U/mL) or heat-aggregated IgG (haIgG) complexes for 45 at 37 °C. HaIgG -complexes were prepared as previously described [40]. Briefly, 10 mg/mL of human IgG was aggregated at 62 °C in PBS for 20 min. Insoluble aggregates were spun out by centrifugation at 5.000 g for 10 min. The soluble fraction corresponds to haIgG-complexes. Finally, the supernatants were collected following centrifugation at 5.000 g for 10 min at 4 °C and stored at −70 °C prior to sCD40L measurements by ELISA as described earlier [41].

### 3.11. Statistical Analysis

Three or more independent experiments were performed. The data were analyzed and expressed as mean ± standard error of mean (SEM) using SPSS version 17.0. The data were statistically analyzed by student paired and one-way analysis of variance using Tukey’s post-hoc test. The statistical significance level was set up at *p* < 0.05.

## 4. Conclusion

In this study, we have demonstrated for the first time that guanosine possesses *in vitro* antiplatelet activity (secretion, spreading, adhesion and aggregation) and inhibition of platelet inflammatory mediator of atherosclerosis (sCD40L). Through bioassay-guided on the basis of antiplatelet activity, was possible identify and isolate a bioactive compound from *S. lycopersicum* with antiplatelet activity. The most important findings of this study suggest that guanosine can markedly inhibit agonist-induced platelet activation. The findings from our study enable a better understanding of guanosine, which could ultimately lead to the development of novel pharmaceutical strategies for the treatment of CVD. Moreover, *S. lycopersicum* may constitute a functional ingredient adding antiplatelet activities to processed foods.
